# Sensitive Simultaneous Measurement of Metformin and Linagliptin in Plasma Samples by Couple of Nano Graphene Oxide-based Dispersive Solid Phase Extraction Method and Liquid Chromatography

**DOI:** 10.22037/ijpr.2019.111659.13292

**Published:** 2020

**Authors:** Ali Gholami, Fahimeh Bahrami, Mohammad Faraji

**Affiliations:** a *Department of Analytical Chemistry, Faculty of Science, University of Kashan, Kashan, Iran. *; b *Department of Food Science and Technology, Faculty of Food Industry and Agriculture, Standard Research Institute (SRI), Karaj, Iran.*

**Keywords:** Dispersive solid phase extraction, HPLC, Plasma analysis, Linagliptin, Metformin, Nano graphene oxide

## Abstract

A simple, rapid, and ultra sensitive dispersive solid phase extraction based on nano graphene oxide was developed for simultaneous measurement of trace amounts of metformin (MET) and linagliptin (LIN) in plasma samples by HPLC-UV-Vis. Affecting factors on the extraction of these drugs, including adsorbent weight, extraction time, organic solvent type, desorption situations, and composition of solvent were examined and optimized. In optimum conditions, the LOD (limit of detection) and LOQ (limit of quantification) of the suggested technique were 2.0 ngmL^-1^ and 6.1 (ngmL^-1^) for LIN and 3.0 ngmL^-1 ^and 9.2 ngmL^-1 ^for MET, respectively. Suitable linear behavior in the considered ranges of concentration (10-2000 ngmL^-1^) and good correlation coefficient of 0.9901 and 0.9903 (r^2^) for LIN and MET were obtained, respectively. The RSD (relative standard deviations) according to three replicate measurements at 2, 20, 200 ngmL^-1 ^levels of these drugs was less than 8.0%. In the last step, applicability of the suggested technique was examined by analyzing the drugs in plasma samples and reasonable results were achieved.

## Introduction

Type II diabetes mellitus affects more than 180 million people in the worldwide and especially in the modern countries which the incidence of the disorder is increasing. This type is defined via multiple metabolic abnormalities including impairing insulin secretion, increasing glucose production, and insulin resistance. Morbidity and mortality associated with type 2 diabetes mellitus is caused by macro vascular complications such as cardiovascular disease and microvascular complications such as retinopathy, neuropathy, and nephropathy. In addition to diet and exercise, a number of medications are available to lower blood sugar levels (1-6). 

**Figure 1:**
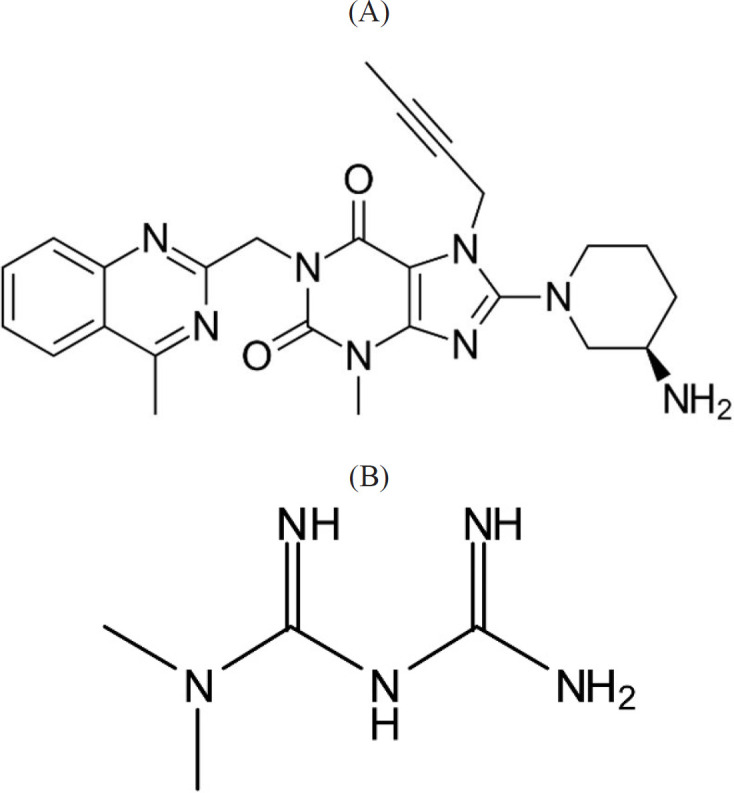
Chemical structure of (A) Linagliptin and (B) Metformin

Linagliptin (LIN) (Figure 1A), novel antidiabetic agents, is utilized with exercise, diet and occasionally with other medicines in type 2 diabetes patients. The basis of its work (when it is high) increases the volumes of specific natural substances that lead to lower blood sugar. LIN is an inhibitor of dipeptidylpeptidase‐4 which degrades the incretion hormones GIP (glucose-dependent insulin tropic polypeptide) and GLP-1 (glucagon-like peptide-1) (7-11).

Metformin HCl (MET) (Figure 1B), a type 2 diabetic drug, is applied as standard fist-line pharmacotherapy which help diabetics to respond to insulin. Similar to common diabetic drugs, Metformin tries to reduce blood sugar to a normal level and keep this level. It is possible to employ Metformin in combination with other drugs of diabetic; moreover, diabetics are advised to have exercise and diet to manage their condition (12-14).

Recent years, the application of nano materials in extraction processes, as adsorbents (stationary phases), has gone through rapid growth (15, 16). Since discovery of Graphene (G) in 2004, it has been surveyed world-wide for various goals, because of its excellent chemical and physical characterization (17-21). The plane of atoms of carbon in graphite oxide (GO) is greatly ornamented by the groups which contain oxygen atom, but the layered structure of graphite and GO is the same. Consequently, Graphene (G) and Graphene oxide (GO) has attracted consideration from many investigators (17).

Review of literature shows that several LC-MS/MS and HPLC techniques have been used alone or combined with other drugs to MET analyze (21-23). But, few HPLC techniques have been applied for simultaneous measurement of MET with LIN (24-30). 

The main goal of this study was developed as a solid phase extraction technique based on nano Graphene oxide for the extraction and enrichment of LIN and MET from plasma samples (for the first time). At the first, affecting parameters on extraction of LIN and MET were estimated and optimized and then, the individual properties of the suggested technique were compared 

with recent published investigates. At the last step, the suggested technique was applied for the measurement of these drugs in the plasma samples.

## Experimental

*Materials and reagents*

All of the used reagents were in analytical grade. LIN and MET were purchased from Alhavi (Tehran, Iran). The chemical structures of these drug are shown in Figures 1A and 1B. Acetonitrile (HPLC-grade), methanol (HPLC-grade), sodium hydroxide, hydrochloric acid, and sodium dilydrogen phosphate were generally provided from Merck. Nano Graphene Oxide (NGO) were purchased from Company of Sigma Aldrich. The diameter of NGO was less than 50 nm. Stock solutions of LIN and MET standards (1000 ngmL^-1^) were provided via dissolving in 5mL methanol and then diluted with reagent water. The working solutions were provided via appropriate dilution of the solution of the standards with water.

*HPLC instrument and procedure*

The mode of HPLC-UVD operating was isocratic, the temperature of column was adjusted to the room temperature and volume of injections were 20 μL. The method was developed on a LiChrosphere 100 RP 18e (125 mm × 4.0 mm, 5 μm) column sustained at an ambient temperature. The combination of mobile phase was mixture of potassium dihydrogen orthophosphate (0.05 M) and methanol in ratio equal to 70:30 (V/V) (pH 4.6 set with ophosphoric acid) which was carried at a 0.6 mL/min flow rate of. The temperature of column was sustained at 25 °C and the detect wavelength was set at 267 nm (7, 28 and 31). The volume of injections was 20 μL. The mobile phase has been filtered with a pore size filter (0.45 µm) and separated via vacuum.

 Ultrasonication of samples was carried out via a 40 kHz (0.138 kW) temperature control ultrasonic water bath. pH measurements were carried out by a Jenway model 3320 pH meter. A Stuart.

*DSPE procedure*

For DSPE process, 25 mg of GO was transferred into a tube and dispersed in 50 mL sample. Then the tube was sonicated for about 2 min and shaken for 10 min. Subsequently, the sorbent was separated and the supernatant was easily discarded. The pre-concentrated target analytes were eluted from the isolated sorbent with 2.0 mL (2 × 1 mL) acetic methanol for about 5 min and eluted analytes collected into a 10-mL screw cap glass test tube and evaporated under the stream of nitrogen. Consequently, after re-dissolving the residue in 100 µL of mobile phase, the solution was introduced to HPLC-UVD for quantification. All experiments were run in triplicates, and the mean values were exploited for optimization.

*Preparation of samples*

For investigating of practicability of the proposed DSPE technique to extract and determine LIN and MET in the real samples, the proposed technique was used to extract these drugs in plasma. Iranian Blood Transfusion Organization provided the study with the plasma samples. Before taking any measures, the plasma samples were put for centrifuging for 5 min until the deposition of all solids. After it, the supernatant was moved to a clean tube. Human plasma samples were gained from healthy males (three man). Standard addition technique was used to measure these drugs in samples. To reduce the effect of matrix, the samples of plasma were diluted 1:10 via ultra-pure water. Therefore, an extra step of preparation was carried out to elimination proteins of plasma by adding 0.5 g to 50 mL of trichloro acetic acid to the diluted sample accomplished by mixture centrifuging for separation of the precipitated proteins. Following the proteins isolation, the steps of extraction were done on clear supernatant solution based on the procedure of DSPE.

## Results and Discussion

*Optimization of DSPE conditions*

Based on preliminary studies in this field, four key parameters were chosen for studying the impacts of the influencing parameters on the selected drugs extraction effectiveness. The variables including desorption, sorbent mass and desorption solvent composition, absorption time and volume were assessed. Some other parameters and their interactions were neglected and were kept for further studies (4, 10).

*Optimization of the sorbent mass*

**Figure 2:**
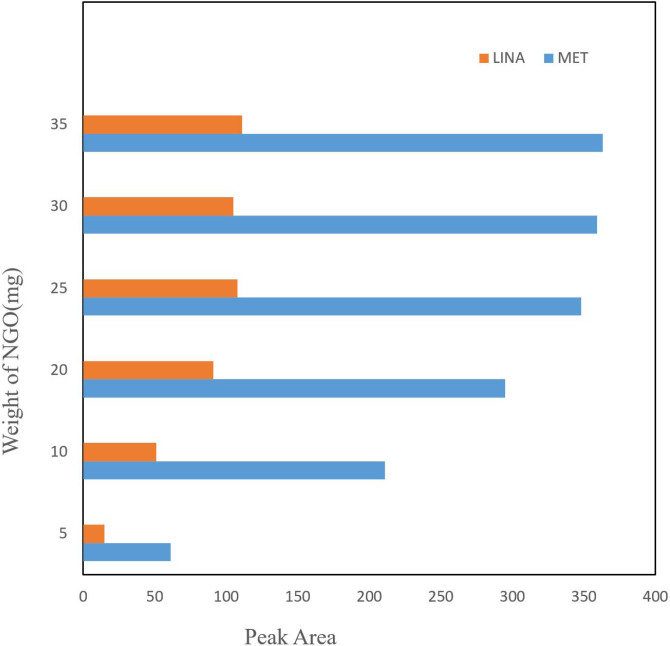
The effect of NGO amounts on efficiency of extraction. Conditions: volume of sample = 50 mL; concentration of the LIN and MET = 100 ngmL^-1^; stirring time = 10 min; elution with 3 mL (2 × 1.5 mL) methanol; desorption time = 7 min

Amount of adsorbent seems to be effective on drug extraction recovery, thus the quantity of NGO was optimized in the 5-35 mg range (Figure 2). The experiment shown that with increasing the amount of adsorption, the yield of extraction continuously improved and reached to equilibrium at 25 mg. Although increasing the adsorbent amounts up to 25 mg possibly contributed to the reaction between analyte and adsorbent by preparing an suitable surface area for adsorption of drug, in higher NGO amounts, low efficiency of extraction was observed. This could be due to NGO accumulation that decreased the surface area of operative adsorption. Subsequently, the following investigations were done by 25 mg of adsorbent.

*Selection of desorption solvent composition and volume*

**Figure 3:**
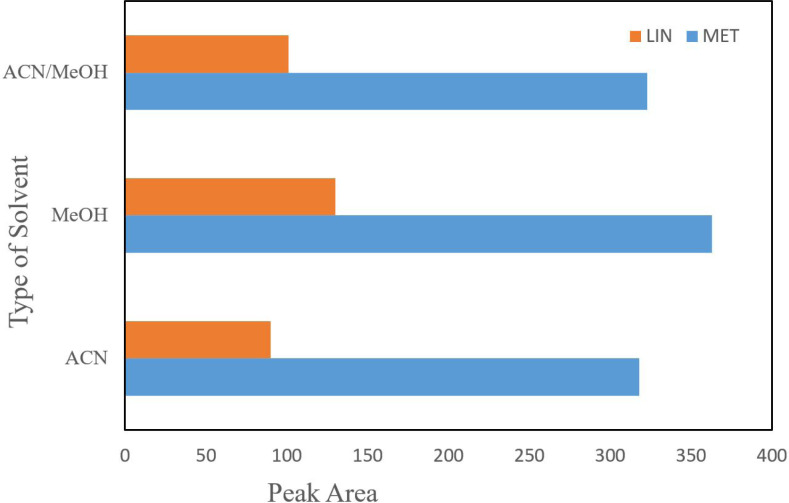
The effect of type of extraction solvent composition and volume on extraction efficiency. Conditions: weight of NGO = 25 mg; Sample volume = 50 mL; concentration of the drugs = 100 ngmL^-1^; stirring time = 10 min; desorption time = 7 min

Various organic solvents including methanol, acetonitrile, and equal mixture of acetonitrile and methanol were tested for removing the analyte from NGO. According to the results, acetic methanol showed the best peak area in comparison with the others and thus acetic methanol was chosen for the following assays. Acetonitrile is a weaker polar solvent than methanol; however, due to the existence of polar functional groups on surface of NGO, it could be appropriately distributed in polar solvents. In fact, most high distribution of NGO in methanol which providing the maximum surface interaction for drug and adsorbent, maximum extraction efficiency can be carried out (Figure 3).

*Effect of adsorption and desorption time*

Since the maximum efficiency of extraction is based on the extraction time to reach equilibrium, the extraction time is a significant parameter in the DSPE method. A range of time between 1 to 15 min was tested for spiked drugs in real samples. Thus, the highest peak area was achieved in 10 min and the efficiency remained constant with subsequently increases in time extraction. The cause of this phenomenon is an expression of equilibrium attainment of extraction in 10 min. 

**Figure 4:**
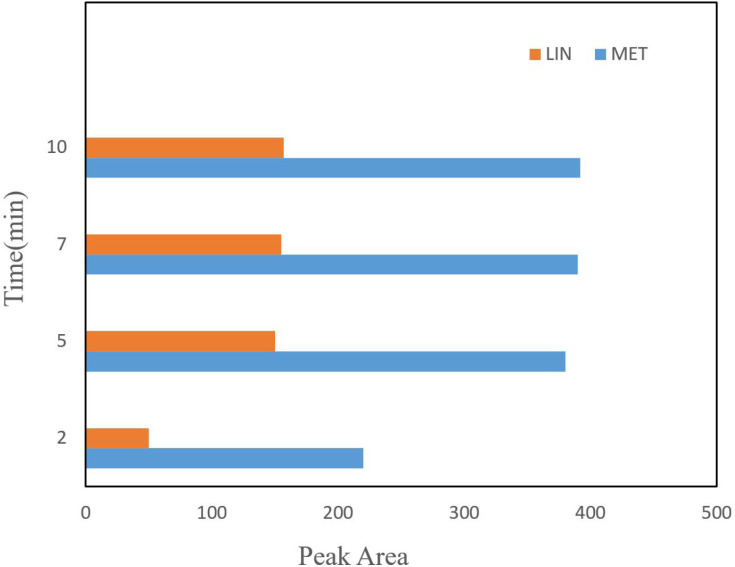
The effect of different desorption time, other extraction conditions are as cited in Figure 3

Time of desorption is another important parameter in the DSPE procedure which influence the mass of desorbed drug from the NGO areas. The amount of desorbed drug was studied in a time range between 2 to 20 min. According to the results, the extraction efficiency increased with an increase in the time up to 5 min, and then the peak area decreased (Figure 4). Therefore, 5 min was selected for the following experiments.

LOD values for each compound was determined according to the S/N = 3 criterion and the LOQ of the assay was assessed also according to the S/N = 10 criterion. Various concentrations of LIN and MET (2, 20, 200 ngmL^-1^) were spiked in blank plasma samples and analyzed by implementing NGO-DSPE-HPLC-UV-Vis technique. Accuracy and precision experiments were done at three concentrations involving the calibration range.

*Analytical parameters*

The quality figures were measured under optimized conditions of extraction. Several quantitative factors such as the dynamic linear range, LOD (limit of detection), correlation coefficient, LOQ (limit of quantification), and RSD (relative standard deviation) were examined to validate the proposed technique (Table 1). Different ngmL^-1 ^concentrations ranging from 2-200 ngmL^-1 ^was spiked in the blank plasma sample and tested using the proposed method. The curve of calibration was created by plotting the average of peak area in comparison to concentration and the correlation coefficient was calculated. Values of LOD for all compounds was measured according to the S/N = 3 criterion and the assay LOQ was assessed according to the S/N = 10 criterion. Different concentrations of LIN and MET (2, 20, 200 ngmL^-1^) were spiked in blank plasma samples and analyzed using NGO-DSPE-HPLC-UV-Vis method. Precision and accuracy of experiments were carried out at three concentrations, covering the range of calibration (Table 2).

*NGO- DSPE -HPLC-UV-Vis applied to real sample*

**Figure 5:**
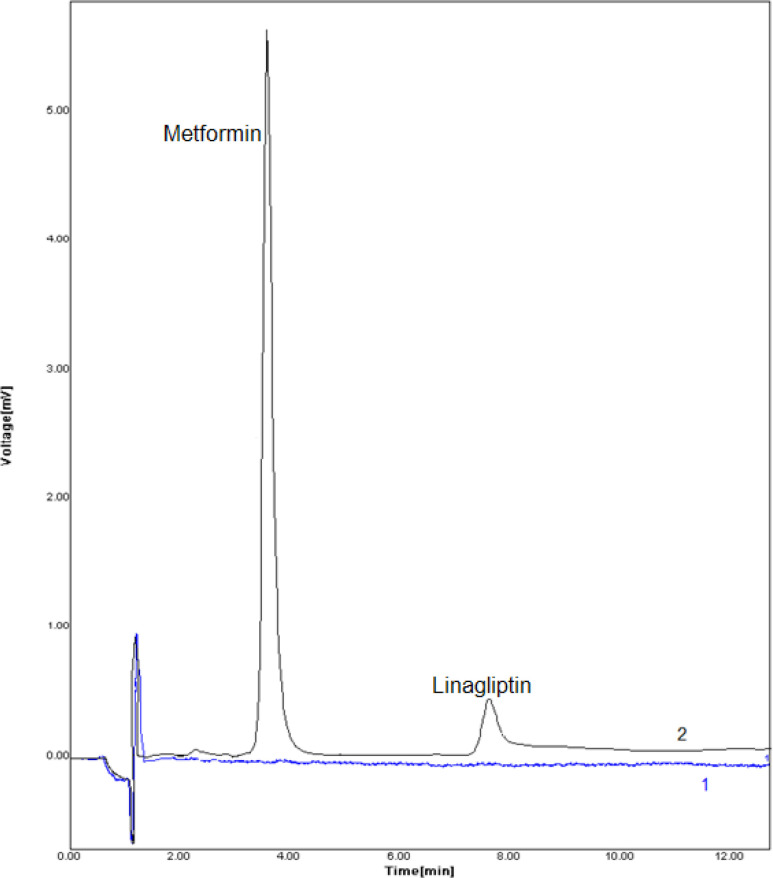
Chromatograms of blank positive plasma samples. Typical chromatograms (HPLC) of (1) blank human plasma, (2) plasma sample spiked with drugs at concentration of 20.0 ngmL^-1^. Conditions: column LiChrosphere 100 RP 18e (125 mm × 4.0 mm, 5 μm) maintained at an ambien temperature, mobile phase methanol: phosphate buffer (potassium dihydrogen orthophosphate 0.05 M pH 4.6) (70:30 v/v) at flow rate of 0.6 mL min^−1^, column temperature: 25 °C, monitoring wavelength: 267 nm, injection volume: 20 μL

The proposed method was used to drug extraction from the sample of plasma to investigate the possibility of developed method to extraction and measurement of LIN and MET in real sample (Table 3). The implementation of the suggested technique was studies using positive plasma samples. Figure 5 displays the DSPE–HPLC–UV-Vis chromatogram in blank and positive plasma sample. Considering that these drugs were not detected in the plasma samples, ngmL^-1^concentrations of them were added into the samples of plasma, and process of extraction and measurement was performed according to the proposed method. 

As Table 2 shows, the experiment results of analyses of plasma sample by the suggested technique were in agreement with the spiking amounts. Moreover, relative errors were acquired less than 4.3% and 5.4% for MET and LIN, in the respective order and suggested technique showed great reproducibility for measurement of these drugs in the plasma samples with intra-day values of RSD% in the range of 3.9-6.1 and 4.5-7.9 for MET and LIN, in the respective order.

*Comparison of proposed methods with previously published methods*

Few researches have focused on determining the selected drugs in plasma matrices concurrently (24, 26 and 28). No result has been yet published on applying DSPE to determine LIN and MET. Table 4 displays the suggested technique based on adsorbent NGO with DSPE pretreatment established comparable detection limits for the major of analytes.

**Table 1:** Table of figures of merit for NGO-based DSPE extraction of LIN and MET

**RSD (%)**	**LOQ (ngmL** ^-1^ **)**	**LOD (ngmL** ^-1^ **)**	**Linearity (r²)**	**Concentration range (ngmL** ^-1^ **)**	**Analyte**
3.2 3.6	6.1 9.2	2.0 3.0	0.9901 0.9903	10-2000 10-2000	Linagliptin Metformin

Linearity is described by the correlation coefficient for the calibration curve.

(LOD): S/N = 3. (LOQ): S/N = 10.

**Table 2:** Result of method validation of proposed method

**Metformin**			**Linagliptin**			**Analyte**
200	20	2	200	20	2	Concentration (ngmL^-1^)
4.5	5.8	7.9	3.9	4.5	6.1	Intra-day (n = 3) Precision (RSDª)
3.7	4.1	5.3	2.7	3.5	4.2	Intra-day (n = 3) Accuracy (bias)
1.0	0.8	0.9	1.1	0.9	0.8	Inter-day (n = 3) Precision (RSDª)
1.1	0.6	0.8	1.0	0.8	0.7	Inter-day (n = 3) Accuracy (bias)

ªRelative standard deviation.

**Table 3:** Result of method validation of NGO-based DSPE method

**Analyte**	**Recovery (%)**
Subject 1	96.23 for Linagliptin 94.33 for Metformin
Subject 2	97.11 for Linagliptin 95.03 for Metformin

**Table 4:** Comparison of different method used for simultaneous determination of LIN and MET

**Analyte**	**Extraction technique**	**Determnation technique**	**Matrix**	**LOD**		**Ref.**
LIN MET	-	RP-HPLC	Pharmaceutical dosage		0.09 µgmL^-1^ 0.06 µgmL^-1^	(24)
LIN MET	-	Spectrophotometric	Pharmaceutical dosage		0.23 µgmL^-1^ 0.77 µgmL^-1^	(26)
LIN MET	-	RP-HPLC	Pharmaceutical dosage		Not recorded Not recorded	(28)
LIN MET	NGO- DSPE	RP-HPLC	Plasma		2 ngmL^-1^ 3 ngmL^-1^	This work

## Conclusion

In the current study, a novel NGO-DSPE-HPLC-UV-Vis procedure was established and confirmed for the simultaneous measurement of LIN and MET in plasma samples. The samples were initially extracted via the NGO-based DSPE, and then the eluents of this stage were exploited for more enrichment and purification of the analytes before analysis of HPLC. The results confirmed the high efficiency of extraction of the method leading to a low detection limit. This method removed biological matrix endogenous interferences, and allowed analysis including easy preparation of the sample and, introduced a sensitive, simple and inexpensive technique to extraction and assessment of the presence of these drugs in positive plasma samples. 

The findings of the present study show that because of the commercial availability of NGO, the suggested techinque would be an effective potential for pre-concentration and determination of target drugs from real samples in the similar way. Moreover, the suggested method not only might offer diagnostic and clinical laboratories with an enhanced analytical technique for ultra-trace assessment of the existence of LIN and MET in another matrix but also it could be used to determine the other Gliptins.
